# Characteristics of diabetic foot ulcers in Western Sydney, Australia

**DOI:** 10.1186/s13047-014-0039-4

**Published:** 2014-09-28

**Authors:** Norafizah Haji Zaine, Joshua Burns, Mauro Vicaretti, John P Fletcher, Lindy Begg, Kerry Hitos

**Affiliations:** Arthritis and Musculoskeletal Research Group, The University of Sydney, Sydney, NSW Australia; Foot Wound Clinic, Department of Surgery, The University of Sydney, Westmead Hospital, Sydney, NSW Australia; Westmead Research Centre for the Evaluation of Surgical Outcomes, Department of Surgery, The University of Sydney, Sydney, NSW Australia

**Keywords:** Diabetes, Diabetic foot ulcer, Socioeconomic

## Abstract

**Background:**

Australia is ranked ninth of 39 countries in the Western Pacific region most affected by diabetes. Patients with diabetes are at high risk of developing foot ulcerations that can develop into non-healing wounds. Recent studies suggest that the lifetime risk of developing a diabetic foot ulcer is as high as 25%. Few studies have reported the prevalence of, risk factors and socioeconomic status associated with, diabetic foot ulcers in Australia. The aim of this study was to evaluate the characteristics of diabetic foot ulcers in a tertiary referral outpatient hospital setting in Western Sydney, Australia.

**Methods:**

From January-December 2011, a total of 195 outpatients with diabetes were retrospectively extracted for analysis from the Westmead Hospital’s Foot Wound Clinic Registry. Data on demographics, socioeconomic status, co-morbidities, foot ulcer characteristics and treatment were recorded on a standardised form.

**Results:**

Demographics and physical characteristics were: 66.2% male, median age 67 years (IQR: 56–76), median body mass index (BMI) of 28 kg/m^2^ (IQR: 25.2-34.1), 75.4% had peripheral neuropathy and the median postcode score for socioeconomic status was 996 (IQR: 897–1022). Diabetic foot ulcer characteristics were: median cross sectional area of 1.5 cm^2^ (IQR: 0.5-7.0), median volume of 0.4 cm^3^ (IQR: 0.11-3.0), 45.1% on the plantar aspect of the foot, 16.6% UT Wound Grade of 0C to 3C (with ischaemia) and 11.8% with a Grade 0D to 3D (with infection and ischaemia) and 25.6% with osteomyelitis. Five patients required an amputation: 1 major and 4 minor amputations.

**Conclusions:**

In accordance with other international studies, foot ulcers are more likely to present on the plantar surface of the foot and largely affect overweight older males with a long standing history diabetes in our outpatient hospital in Western Sydney.

## Background

Australia is ranked ninth of 39 countries in the Western Pacific region most affected by diabetes [1,2]. People with diabetes have a 25% lifetime risk of developing foot ulcers, which can develop into chronic non-healing ulcers [3,4]. Chronic ulcers often contain bacterial biofilms that can lead to chronic infections [5]. Foot ulcers may develop due to overlapping factors including neuropathy, peripheral arterial disease, pressure overload, trauma and foot pathologies such as fissures and callosities. Approximately 40-70% of lower limb amputations are diabetes-related and 85% are preceded by foot ulceration [6].

At any point in time, it has been reported in Europe, USA and Africa that 1-11% of the population with diabetes have an active foot ulcer [7–10]. In the largest database of foot ulcers in Australia [11] , Lazzarini *et al.* examined the characteristics of ambulatory patients with a foot ulcer across 13 Health and Hospital Services and reported that of 2,034 people presenting with a foot ulcer, 85% had a history of diabetes. The ANDIAB (Australian National Diabetes Information Audit & Benchmarking) Report of adults with diabetes found that 1.7% had a current foot ulcer in 2009 which increased to 2.1% in 2011 [12,13]. The large EURODIALE (European Study Group on Diabetes and the Lower Extremity) Project across 14 European centres in 2003 to 2004 [14], identified peripheral arterial disease (PAD) and peripheral neuropathy as the major risk factors in the development of diabetic foot ulcers [15].

The Australian Diabetes Society recommends that people with a foot ulcer are cared for by a multidisciplinary team [16]. The Westmead Hospital’s Foot Wound Clinic is an interdisciplinary clinic which is attended concurrently by podiatrists, vascular consultants and registrars, wound care consultants, vascular clinical nurse consultants and a clinic nurse. Infectious Disease Consultants are also available upon request [17]. The Australian National Evidence-Based Guidelines on foot complications in diabetes highlights the importance of understanding the characteristics of an ulcer to help monitor the response of an ulcer to treatment [18]. There have been no studies reporting primarily on characteristics of, risk factors and socioeconomic status associated with, diabetic foot ulcers in Western Sydney, Australia.

The primary aim of this study was to evaluate the characteristics of diabetic foot ulcers in patients presenting to a tertiary referral outpatient hospital setting in Western Sydney, Australia. The secondary aim was to evaluate the use of vascular investigations and off-loading modalities in this high risk group of patients.

## Methods

Ethical approval was granted from the Research Ethics Committees at the Western Sydney Local Health District and The University of Sydney. The study population was defined as the total number of patients with Type 1 Diabetes Mellitus (Type 1 DM) and Type 2 Diabetes Mellitus (Type 2 DM) with foot ulcers at initial visit attending our outpatient Foot Wound Clinic at Westmead Hospital between January to December 2011. Diabetes Mellitus was defined according to the criteria set by the World Health Organisation (WHO). The current WHO diagnostic criteria for Diabetes Mellitus includes a fasting plasma glucose ≥ 7.0 mmol/l (126 mg/dl) or 2-hour plasma glucose ≥ 11.1 mmol/l (200 mg/dl) [19]. A foot ulcer was defined as a full-thickness wound located distal to the ankle (level of malleoli) [20].

All data were captured in Westmead Hospital’s Wound Clinic Registry. Data were extracted on a standardised data collection form. For inconsistencies, clarification was sought from the treating clinician or the patient medical record. Patients without diabetes and without foot ulcers were excluded from the study. Background data included patient characteristics such as demographics details, socioeconomic factors, marital status, country of birth and English language status (defined as patients who were English and non English speaking). Other patient characteristics included co-morbidities such as neuropathy, hyperlipidaemia, retinopathy and history of amputation and/or ulceration.

Peripheral neuropathy was diagnosed by a Podiatrist using a neurothesiometer, 128Hz tuning fork or 10 g monofilament. Investigations of diabetic foot ulcer related factors (such as PAD and ulcer infection), referrals to other health professionals, treatments (such as pressure offloading) and hospitalisation and/or requiring vascular or surgical interventions were also recorded. PAD was assessed and diagnosed by measuring toe pressures using a photoplethysmography (Hadeco Smartdop 30 EX Vascular Ultrasound Doppler). A toe pressure of <55 mmHg indicates PAD in a foot [21,22].

The socioeconomic status was based on the Australian Bureau of Statistics (ABS) residential postcode method for the general Australian population (mean index = 1000) [23]. The Index of Relative Socioeconomic Disadvantage (IRSD) is used by the ABS as a general socio-economic index to summarise a range of information about the economic and social conditions of people and households within an area. A low score indicates relatively greater disadvantage whereas a high score indicates relatively advantage in general [24]. A score of less than 1000 indicates that the area is more disadvantaged than the average area at the Statistical Area Level 1 (SA1). SA1 is the smallest geographical unit at which the SEIFA (Socio-Economic Indexes For Areas) indexes are calculated [25].

Information on osteomyelitis, Charcot foot and diabetic foot ulcer PEDIS grades of infection (skin/subcutaneous), size, location, infection, history of previous ulceration and lower extremity amputation were recorded [26]. According to the PEDIS classification, grades of infection were defined as, Grade 1: No symptoms or signs, Grade 2: Inflammation of skin/subcutaneous tissues only, Grade 3: Extensive erythema deeper (>2 cm) than skin/subcutaneous tissues and Grade 4: Systemic inflammatory response syndrome) [26]. The probe to bone technique was used to diagnose osteomyelitis in diabetic foot ulcers [27]. The University of Texas (UT) Diabetic Wound Classification System was used to classify diabetic foot ulcers into a single validated grading system [28].

Foot ulcer duration was categorised into <1 week, 1 week to 3 months and >3 months [14]. In our study, re-ulceration was used to define a previous foot ulcer that has re-ulcerated on the same location. A patient with a history of a foot ulcer was defined as a patient who had an ulcer on any location of either foot. If more than one ulcer was present, the primary ulcer was defined by the ulcer with the largest cross sectional area (cm^2^) [15,20]. UT Wound Classification of 0A and 0C are considered completely epithelialized [18]. A traumatic event was defined as an acute injury such as a footwear rub, blister or plantar pressure overload.

### Statistical analysis

Descriptive statistics to characterise the study sample were generated using SPSS 21.0 (IBM SPSS Statistics for Windows, Armonk, NY, USA). Normality of data distribution was assessed using the Kolmogorov–Smirnov test with Lilliefors significance correction. As such, continuous data are presented as median and interquartile range (IQR, 25^th^ and 75^th^ quartiles). Continuous data were compared using the Mann Whitney U test and proportions using the chi squared (χ^2^) test. All inferential tests were two tailed and statistical significant differences were considered at the P < 0.05 level.

## Results

### Patient demographics, risk factors and co-morbidities

Overall, 318 patients were initially extracted from Westmead Hospital’s Wound Clinic Registry and data from 195 (61.3%) patients with a diabetic foot ulcer at their initial visit were analysed. The remaining 123 (38.4%) patients were excluded due to no history of diabetes.

Demographics and patient characteristics are shown in Table 1. The male to female ratio was 2:1, with a male median age of 65 years (IQR: 56–76) and a female median age of 69.5 years (IQR: 56–76) (P = 0.154). Of 165 (85%) patients with diabetes duration data, 9.1% were <5 years, 11.5% were 5–10 years and 79.4% were >10 years duration. More than 70% of patients with BMI data (n = 121) were overweight or obese (BMI ≥ 25 kg/m^2^). A total of 96 (49%) patients were born overseas and were generally (88.8%) English speaking individuals. The two most prevalent co-morbidities were neuropathy (75.4%) and hypertension (67.2%). Over 50% of patients with diabetic foot ulcers were smokers or ex smokers. A total of 41.5% of patients had a history of foot ulcer on another location on the foot. The complete list of medical history and lifestyle risk factors are shown in Table 2.

**Table 1:** **Demographics of the sample**

**Variable**	**Total participants**
Age (median years, IQR^‡^), n = 195	67 (56–76)
Gender, Male, no. (%), n = 195	129 (66.2%)
Height (median metres, IQR^‡^), n = 129	1.7 (1.6-1.8)
Weight (median kg, IQR^‡^), n = 132	84.5 (70.3-101.0)
BMI (median kg/m^2^, IQR^‡^), n = 122	28 (25.2-34.1)
BMI category*, no. (%), n = 121	
Underweight	4 (3.3%)
Normal	23 (19%)
Overweight	43 (35.5%)
Obese	36 (29.8%)
Morbidly Obese	15 (12.4%)
Socioeconomic^#^ (median score, IQR^‡^), n = 193	996 (897–1022)
Nationality, no. (%), n = 195	
Australian born	99 (50.8%)
Born overseas	96 (49.2%)
Marital Status, no. (%), n = 195	
Married or De Facto	114 (58.5%)
Widowed	25 (12.8%)
Single	55 (28.2%)
Other	1 (0.5%)
Duration of DM (median years, IQR^‡^), n = 165	17 (11–25)

*Overweight was defined as BMI 25.0-29.9 kg/m^2^; Obese was defined as BMI 30.0-39.9 kg/m^2^; Morbidly Obese was defined as BMI > 40.0 kg/m^2^.

^#^Australia Bureau Statistics postcode score.

^‡^IQR: 25^th^ to 75^th^ percentile.

**Table 2:** **Medical history and lifestyle risk factors of the sample (n = 195)**

**Factor**	**Number (%)**
Neuropathy	147 (75.4%)
Hypertension	131 (67.2%)
Hyperlipidaemia	107 (54.9%)
History of ulcer (Healed)	81 (41.5%)
Retinopathy	77 (39.5%)
History of amputation	64 (32.8%)
Angina/Infarct	47 (24.1%)
Nephropathy	43 (22.1%)
Renal Failure	26 (13.3%)
Claudication	22 (11.3%)
Cerebrovascular Accident	21 (10.8%)
Transient Ischaemic Attack	15 (7.7%)
Charcot Arthropathy	11 (5.6%)
Smoking	
Smoker	28 (14.5%)
Ex smoker	83 (42.6%)

The median socioeconomic index score was 996 (IQR: 897–1022) which ranked in decile 6 and in the 51^st^ percentile for Australia (Table 1). Of the 82 (42.1%) patients with a diabetic foot ulcer from relatively advantaged areas (IRSD score >1000), 31 (38%) had a history of ulceration and 23 (28%) had a history of amputation. Of the 113 (57.9%) patients from relatively disadvantaged areas (IRSD score of <1000), 49 (43%) had a history of ulceration and 41 (36%) had a history of amputation. There was no significant difference between history of ulceration (P = 0.367) and amputation (P = 0.227) in IRSD scores.

### Foot ulcer characteristics

In total, 96.4% of diabetic foot ulcers were recorded as new ulcers during the initial visit and seven (3.6%) were recorded as reulcerations. Of the 195 patients with diabetic foot ulcers, more than a third (35.4%) had multiple ulcers. Primary ulcer characteristics and UT Wound Classifications are shown in Tables 3 and 4 respectively. The median cross sectional area of the primary ulcer was 1.5 cm^2^ (IQR: 0.5-7.0 cm^2^) and volume was 0.4 cm^3^ (IQR: 0.1-3.0 cm^3^). Ulcer cross sectional area was <1 cm^2^ in 42% of cases, between 1 and 5 cm^2^ in 30% of cases, and >5 cm^2^ in 28% of cases. Over one third (36.4%) of foot ulcers were located on the forefoot and over 45.1% were located on the plantar aspect of the foot. The duration of the ulcers at initial visit was < 1 week for one patient (0.6%), 1 week to 3 months in (83.4%) of patients and >3 months in 16.0% of patients. The greatest ulcer duration was 208 weeks at initial visit. Predominant UT wound types consisted of category 1A (33.7%), 1B (14.0%) and 3B (11.9%) (Table 4).

**Table 3:** **Primary ulcer characteristics of the sample**

**Characteristic**	**Total participants**
Anatomical Region, n = 195	
Hallux, no. (%)	41 (21%)
Digits, no. (%)	29 (14.9%)
Forefoot, no. (%)	71 (36.4%)
Midfoot, no. (%)	16 (8.2%)
Heel, no. (%)	38 (19.5%)
Location, n = 195	
Plantar, no. (%)	88 (45.1%)
Dorsal, no. (%)	29 (14.9%)
Lateral, no. (%)	27 (13.8%)
Medial, no. (%)	21 (10.8%)
Apex, no. (%)	30 (15.4%)
Side, n = 195	
Right, no. (%)	95 (48.7%)
Left, no. (%)	100 (51.3%)
Duration (weeks), n = 169 median (IQR*)	6 (3–16)
< 1 week, no. (%)	1 (0.6%)
1 week – 3 months (12 weeks), no. (%)	141 (83.4%)
> 3 months (12 weeks), no. (%)	27 (16.0%)
Size	
Length (median cm, IQR*), n = 184	1.5 (0.8-3)
Width (median cm, IQR*), n = 184	1 (0.5-2.0)
Depth (median cm, IQR*), n = 183	0.2 (0.1-0.8)
Cross sectional area (median cm^2^, IQR*), n = 183	1.5 (0.5-7.0)
Volume (median cm^3^, IQR*), n = 182	0.4 (0.11-3.0)

*IQR: 25^th^ to 75^th^ percentile.

**Table 4:** **Primary ulcer grade/depth according to The University of Texas Classification System for diabetic foot wounds** [17]

	**Grade/Depth N = 193**				
		**0**	**1**	**2**	**3**
		**Pre- or post- ulcerative lesion completely epithelialised**	**Superficial wound not involving tendon, capsule or bone**	**Wound penetrating to tendon or capsule**	**Wound penetrating to bone or joint**
Stage/Comorbidities N = 193	A	n = 0	n = 65 (33.7%)	n = 5 (2.6%)	n = 3 (1.6%)
	B With infection	n = 0	n = 27 (14.0%)	n = 15 (7.8%)	n = 23 (11.9%)
	C With ischaemia	n = 1 (0.5%)	n = 18 (9.3%)	n = 2 (1.0%)	n = 2 (1.0%)
	D With infection and ischaemia	n = 0	n = 14 (7.3%)	n = 7 (3.6%)	n = 11 (5.7%)

The infection status of each ulcer was graded using the PEDIS system [26]. Almost (49.7%) of all ulcers were infected and the most prevalent was Grade 2 (21.5%) and Grade 3 (26.7%) (Table 5). A total of 50 (25.6%) patients with a diabetic foot ulcer presented with osteomyelitis, and of these 34 (17.4%) were positively diagnosed using the probe to bone technique with 14 (7.2%) confirmed by imaging. The total of 34 foot ulcers diagnosed using probe to bone were also classified using the University of Texas Wound Classification System (Table 4). Approximately 67.7% of ulcers were attributed to traumatic events. The causes of foot ulceration were post surgery (8.2%), traumatic event (67.7%), other (23.6%) and unknown (0.5%).

**Table 5:** **PEDIS classification grades of infection (n = 195)**

**Grades of infection**	**Number (%)**
Grade 1 No symptoms or signs	12 (6.2%)
Grade 2 Inflammation of skin/subcutaneous	42 (21.5%)
tissues only	
Grade 3 Extensive erythema deeper (>2 cm)	52 (26.7%)
than skin/subcutaneous tissues	
Grade 4 Systemic inflammatory response	3 (1.5%)
syndrome	
Missing data	86 (44.1%)

### Pressure off-loading

At the initial visit, the two most commonly prescribed off-loading modalities were the Darco Medical Surgical post-op shoe (29.9%) and Sports/Orthopaedic shoes (15.4%). Three patients (1.5%) were provided with irremovable total contact cast (TCC) and two patients (1.0%) with removable TCC. All TCC (irremovable and removable) were fibreglass casts constructed with 3 M Softcast and Primacast according to our standardised protocol [29]. In total 15% of patients had other types of off-loading modalities which included air mattress for pressure off-loading, 12 mm Poron combination innersole, Forefoot Wedge Shoe and Eggshell Foam Boot.

### Vascular investigations

Thirty-one patients were referred for further vascular investigations. Of these, 16 (52%) were referred for endovascular, 7 (23%) for duplex arterial ultrasound, 6 (19%) for diagnostic angiogram and 2 (6%) for both endovascular and diagnostic angiogram. The predominant UT Wound grades for these 31 patients were 1C (22.6%) and 1D (19.3%).

### Clinical outcome

A total of 5 (2.6%) patients required an amputation after their initial visit (Table 6). Of these, 3 (60%) patients were from a relatively greater disadvantaged area (IRSD score of < 1000) and only one had a history of amputation. Four of the 5 (90%) patients had neuropathy and 3 (60%) were non-smokers. The type and time to amputations are shown in Table 5. There were no deaths during the period of study.

**Table 6:** **Type of amputation and time to follow-up for the sample**

**Patient**	**Time to amputation (in days)**	**Type of amputation**	*IRSD score	**Decile	***Percentile
1	3	Left 4^th^ ray	897	1	9
2	29	Right below knee	820	1	3
	11	Right above knee			
3	13	Left 2^nd^ digit	1034	8	71
4	1	Left ray	820	1	3
5	1	Left 4^th^ digit	1011	7	63

*IRSD: The Index of Relative Socioeconomic Disadvantage.

**The lowest 10% of areas are given decile 1.

***The lowest 1% of areas are given a percentile 1.

## Discussion

This is the first study to report characteristics of diabetic foot ulcers from Westmead Hospital’s Foot Wound Clinic Registry. This may also be the largest study in Australia to date investigating the classification, characteristics, location of diabetic foot ulcers and the patients’ socioeconomic status. Patients with diabetic foot ulcers were predominantly male with long-standing diabetes. A similar retrospective study conducted with 181 patients in Victoria reported 61.3% male predominance [30]. About a third of patients with foot ulcers had a history of ulceration and amputation which are known risk factors for subsequent ulceration [31]. Wu and Armstrong showed that between 20-58% of patients with diabetic foot ulcers develop another ulcer within a year after healing [32].

In our study, three-quarters of patients had a comorbidity of neuropathy which is one of the most common risk factors for developing a diabetic foot ulcer. Over half of patients in this study were smokers or ex-smokers which is a strong risk factor for peripheral arterial disease [33]. The EURODIALE studies highlighted peripheral arterial disease (49%) and neuropathy (86%) as two major risk factors of diabetic foot ulcerations [14].

The median age of our patients was 67 years, which is comparable to the retrospective study conducted in Victoria reporting a mean age of 64.2 years [30]. Increasing age may be a contributory intrinsic factor to chronic wounds as the skin can easily damage [34]. Older cells do not proliferate as fast and may not have an adequate response to stress in terms of gene up regulation of stress-related proteins [34]. Over half of all patients were overweight and obese/morbidly obese which increases the risk of cardiac-related disease and makes offloading more difficult. Obese patients may also experience compromised wound healing due to poor blood supply to adipose tissue [35].

Westmead Hospital has a large catchment area and is culturally diverse with a variable socioeconomic mix [23]. According to the Postal Area (POA) spreadsheet for IRSD, an area code with a score of 996 would be ranked in decile 6 and in the 51^st^ percentile [36]. This indicates that the area is more relatively advantaged than 50% of the areas and more relatively disadvantaged than 49% of areas. Our median socioeconomic index score of 996 reflects a marginally lower than median index score of 1000. There are no studies evaluating the socioeconomic status of patients with diabetic foot ulcers in Australia and whether it is associated with an increased incidence in ulceration. This is the first study exploring the socioeconomic index scores of ambulatory Australian patients diabetic foot ulcer.

Forefoot and digital (including hallux) ulcers were present in 72.3% of patients. This finding is comparable to 77% reported in the EURODIALE study and to a Turkish study investigating predominantly acute and chronic diabetic foot ulcers (78%) [15,37]. Diabetic foot ulcers are commonly located on the plantar aspect of the foot due to abnormal loading and the presence of neuropathy [38]. As such offloading is an important therapeutic option. In the EURODIALE study, 78% of patients received some form of off-loading. Thirty five percent of patients were prescribed a TCC or another casting modality and the majority of plantar ulcers, like our study, were treated with temporary footwear [14]. Interestingly, the low rate (2%) of TCC application in our study was consistent with the rate reported in the USA (1.7%) [39].

The ulcer types were heterogeneous ranging from superficial to deep involving tendon, bone and joint with infection and ischaemia. A total of 11.8% patients had UT Wound Grade 0C to 3C (with ischaemia) and 16.6% Grade 0D to 3D (with infection and ischaemia). Approximately half (49.7%) of our cohort exhibited infected ulcers which is lower than the EURODIALE studies (58%) [15,40]. Fifty patients with a diabetic foot ulcer presented with osteomyelitis. The probe to bone is a quick, low cost and efficient screening test to diagnose osteomyelitis in patients with diabetic foot ulcers [27]. However, other screening methods such as bone biopsy and imaging techniques e.g. computerised tomography (CT) scan, X-ray and magnetic resonance imaging (MRI), can also be used to further confirm the presence of osteomyelitis.

This study is limited by the evaluation of patients with diabetes only. There is also a need to consider other factors such as HbA1c, specifying cause of ulcers due to foot deformities (such as hallux valgus, clawed toes), foot pathologies (such as fissures and callosities) and biomechanical abnormalities (such as cavus and Charcot foot), medical history such as malignancies/chemotherapy and medications which may impair wound healing. Duration of foot ulcer was generally self-reported which is limitation and unreliable for determining ulcer initiation. The Foot Wound Clinic Registry Data Form we used has yet to be assessed for inter-rater reliability and so interpretative errors relating to ulcer characteristics and classification may have occurred. However, to reduce the potential for error, the Foot Wound Clinic Registry includes training in procedures, data sources, data collection systems and most importantly data definitions and their interpretation. A final limitation is that the data reported were derived from a retrospective analysis of a single site and excluded other diabetic foot clinics in Western Sydney.

### Future research

There is a paucity of information on predictive values of risk factors for diabetic foot ulcerations in the Australian health care setting. Therefore in-depth information gained from this study will be useful in developing a risk assessment-model for a larger prospective cohort study. This will enable clinicians to identify and estimate the risk factors associated with diabetic foot ulcers (such as patient co-morbidities, history and physical examination). For comparison, future studies should also evaluate people without diabetes who present with foot ulcers.

## Conclusion

In Western Sydney, diabetic foot ulcers largely affect overweight older males beneath the plantar aspect of the foot with a duration of 1 week to 3 months. These findings are in accordance with the EURODIALE Study. Furthermore our results suggest that socioeconomic status is not related to diabetic foot ulcer characteristics in Western Sydney.

## Abbreviations

ABS: Australian Bureau of Statistics
BMI: Body mass index
DM: Diabetes mellitus
EURODIALE: European Study Group on Diabetes and the Lower Extremity
IQR: Interquartile range
PEDIS: Perfusion, extent/size, depth/tissue loss, infection and sensation
TCC: Total contact cast
UT: University of Texas
